# Seasonality in Human Zoonotic Enteric Diseases: A Systematic Review

**DOI:** 10.1371/journal.pone.0031883

**Published:** 2012-04-02

**Authors:** Aparna Lal, Simon Hales, Nigel French, Michael G. Baker

**Affiliations:** 1 Department of Public Health, University of Otago, Wellington, Wellington, New Zealand; 2 Hopkirk Research Institute, Massey University, Palmerston North, Manawatu, New Zealand; Fred Hutchinson Cancer Research Center, United States of America

## Abstract

**Background:**

Although seasonality is a defining characteristic of many infectious diseases, few studies have described and compared seasonal patterns across diseases globally, impeding our understanding of putative mechanisms. Here, we review seasonal patterns across five enteric zoonotic diseases: campylobacteriosis, salmonellosis, vero-cytotoxigenic *Escherichia coli* (VTEC), cryptosporidiosis and giardiasis in the context of two primary drivers of seasonality: (i) environmental effects on pathogen occurrence and pathogen-host associations and (ii) population characteristics/behaviour.

**Methodology/Principal Findings:**

We systematically reviewed published literature from 1960–2010, resulting in the review of 86 studies across the five diseases. The Gini coefficient compared temporal variations in incidence across diseases and the monthly seasonality index characterised timing of seasonal peaks. Consistent seasonal patterns across transnational boundaries, albeit with regional variations was observed. The bacterial diseases all had a distinct summer peak, with identical Gini values for campylobacteriosis and salmonellosis (0.22) and a higher index for VTEC (Gini = 0.36). Cryptosporidiosis displayed a bi-modal peak with spring and summer highs and the most marked temporal variation (Gini = 0.39). Giardiasis showed a relatively small summer increase and was the least variable (Gini = 0.18).

**Conclusions/Significance:**

Seasonal variation in enteric zoonotic diseases is ubiquitous, with regional variations highlighting complex environment-pathogen-host interactions. Results suggest that proximal environmental influences and host population dynamics, together with distal, longer-term climatic variability could have important direct and indirect consequences for future enteric disease risk. Additional understanding of the concerted influence of these factors on disease patterns may improve assessment and prediction of enteric disease burden in temperate, developed countries.

## Introduction

Seasonality is characteristic of many infectious diseases [1]–[2]. Zoonoses, defined here as those diseases with predominantly animal reservoirs, can cause high morbidity in healthy adults [3] and more serious outcomes in susceptible populations [4], [5]. In temperate, developed countries these diseases exhibit patterns associated with weather and display seasonal peaks alternating with low background levels of infection [6], [7]. The bacterial diseases, campylobacteriosis, salmonellosis and VTEC, peak in summer and decrease in winter [8], [9], [10]. Among protozoan diseases, cryptosporidiosis has a definite seasonality, with spring peaks reported in the United Kingdom and New Zealand and summer-autumn peaks in the United States and Canada [11], [12], [13]. In contrast, giardiasis is markedly less seasonal, albeit with an early autumn peak in some countries [14]. Such regular, recurring patterns indicate a strong, direct, environmental influence on pathogen epidemiology [15], pathogen reservoirs and transmission pathways [16], [17] or factors that affect frequency of pathogen-host interactions [18]. However, such patterns have also been attributed to population characteristics such as seasonal farming [19] and recreational activities [20], mobility patterns [21] and periodic changes in host susceptibility to infection [22]. Thus, large scale environmental influences and associated host demographics are predicted to strongly determine future enteric disease incidence through their effect on host pathogen load, transmission opportunities and spread [23], [24]. To further our understanding of the putative environmental and population associated mechanisms driving enteric disease seasonality, it is helpful to compare seasonal patterns across diseases and regions.

To date, international comparisons of enteric zoonotic diseases have been restricted to a single disease [25], [26] or pathogen groups [27], associations with weather variables [28], [29], other specific risk factors [30], outbreaks [31] or the burden of non-pathogen specific diarrhoeal disease [32], [33]. Multi-pathogen studies are generally limited to detailed analyses of regional data (e.g. [2], [34]). To our knowledge, there exists no compiled evidence documenting seasonality across enteric zoonotic diseases in temperate, developed countries. Such a cross-country, multi-disease approach may be especially informative when integrating potent trans-boundary issues such as human health, population migration and ecological change.

Our review focuses on environmentally mediated, enteric diseases with predominantly zoonotic (nonhuman) reservoirs; group III b in Eisenberg’s *et al.* [35] classification of infectious diseases based on dominant transmission pathways, hereafter referred to as enteric zoonotic diseases. By restricting our review to regions governed by similar climate mechanisms, comparisons of seasonal patterns across countries may facilitate identification of environmental and population influences underlying seasonal variations in disease incidence [36]. Consequently, we confine our study to temperate regions. It is equally important to also account for dominant non-climatic differences among countries that may mask seasonal patterns [37]. We attempt this by limiting our review to OECD (Organisation for Economic Co-operation and Development) member states; countries that have relatively similar economies, public health infrastructure, population dynamics and disease reporting standards.

We assess seasonal patterns in important human enteric zoonotic diseases, notably campylobacteriosis, salmonellosis, VTEC (Vero-cytotoxigenic *Escherichia coli*), cryptosporidiosis and giardiasis among temperate, developed countries and discuss our results in the context of how these patterns could be driven by environmental influences and population characteristics. Specifically, we use a systematic review of the literature and statistical summaries of temporal distribution to establish: (i) the existence (or absence) of a consistent, pattern of disease incidence and quantify the overall magnitude of temporal inequality for each pathogen; and, (ii) compare monthly disease patterns among regions by applying a seasonality index.

## Methods

### Systematic Review

#### Search strategy

The five diseases chosen for review were based on Eisenberg’s *et al.* [35]classification of infectious diseases based on dominant transmission pathways. We focused on environmentally mediated, enteric diseases with predominantly zoonotic (nonhuman) reservoirs; group III b in the classification. Using three electronic databases, PubMed, Web of Science and Embase, we searched publications across the 1960–2010 period that quantified the temporal patterns of campylobacteriosis, salmonellosis, VTEC, cryptosporidiosis and giardiasis in humans. The keywords used were: (“season”, “seasonality” “temporal”), AND (“campylobacteriosis” “campylobacter”, “salmonellosis” “salmonella”, “VTEC” “STEC”, “cryptosporidiosis” “cryptosporidium”, “giardiasis” “giardia”). No language or database restrictions were imposed on the searches. For each disease, citations resulting from the database searches were exported into a master library, and duplicates removed based on parameters of identical author, date and title. Full-text versions of the articles that fulfilled our eligibility criteria were obtained and their reference lists were manually searched to identify any further relevant manuscripts. We also examined the bibliographies of reviews published on pathogen specific epidemiology to identify additional sources for inclusion in the analysis.

#### Study selection

Screening of articles for eligibility consisted of two steps. The first step aimed to exclude papers not relevant to the review and consisted of (i) identifying papers where research was conducted in non-OECD or tropical regions or polar regions (studies conducted outside of 23.5°N–66.5°N and 23.5°S–66.5°S) and, (ii) assessing the residual citations using the question “Does the title, and/or abstract and text explicitly describe data relating to the temporal and/or seasonal variation in the disease of interest in humans?” (adapted from [38]). Here, an explicit description of seasonal variation consisted of text and/or graphics documenting the number or percentage of cases or incidence rates over the study duration.

The second stage involved critically evaluating the studies using the following *a priori* established criteria. Studies were included if (i) they had been conducted continuously for a minimum of a full year to cover all seasons, (ii) the primary outcome was a laboratory confirmed diagnosis of the enteric pathogen of interest, (iii) the study reported case data temporally (day, week, month) (iv) the study was written in English and published in a peer reviewed journal and, (v) study design did not include intervention trials.

Studies that satisfied the inclusion conditions but failed to be representative of the general population were excluded. These comprised studies that were (i) conducted in institutions (e.g. day-care centres, rest homes), (ii) directed at specific demographic groups (e.g. children, elderly) and groups with certain characteristics (immune-compromised, travellers), (iii) studies focussed solely on outbreaks as these studies were generally of a shorter duration and can reflect disease patterns that may not be typical of disease seasonality generally, (iv) studies looking at broader gastro-enteric outcomes (GI) or infectious intestinal disease (IID), (v) studies focussed on the microbiological and immunological characteristics and molecular ecology of the pathogen, (vi) clinical studies, (vi) review articles, although these were used as a source for additional papers.

All data used here were taken from the papers, either directly from the tables or extracted from graphs using DigitizeIt software e.g. [39] or in four cases, by contacting authors. For each pathogen, the relevant studies were examined in detail and the following information recorded: authors and year of publication, location and study duration, study design and sample size, temporal resolution of case data, age range of population, and the timing of the peak number of cases reported.

#### Quantitative data synthesis

Based on geographical and political boundaries studies were assigned to the following regions: United Kingdom (UK) which includes Ireland, Scotland and Wales, Continental Europe which includes studies in mainland Europe, North America (USA), Canada, Oceania (Australia and New Zealand), and the Asian region. As a majority of the data came from countries in the northern hemisphere, data from the southern hemisphere countries were adjusted by six months for the opposite season (i.e. January in the northern hemisphere was aligned with July in the southern hemisphere) [25]. Seasons were defined based on their occurrence in the Northern hemisphere: Winter (December-February), spring (March-May), summer ( June-August) and autumn (September-November).

We assessed disease patterns using the Gini index and a monthly seasonality index. Study-specific monthly averages were obtained by dividing the total number of cases for each month by the number of relevant months in the study. For example, to calculate the average for January over January 1992- December 1995, we took the number of cases in each month, added them together and divided by the number of relevant months in the study (in this case, four). Next, we calculated an annual average for each study by dividing by the number of monthly averages (e.g. January average + February average ……÷12 = annual average). Study-specific monthly averages were divided by the annual average and thus converted to proportions (e.g. January average÷annual average = January proportion) [40]. These proportions were used for the calculation of the Lorenz curve, Gini coefficient and the monthly seasonality index.

#### Lorenz curve and Gini coefficient

In order to characterize seasonality for each disease we developed Lorenz curve models [41] for each disease and determined the corresponding Gini coefficients [42]. In this study, the Lorenz curve is a graphical representation of the cumulative distribution of disease incidence as a function of proportion of the year. If incidence is equally distributed across the year, the Lorenz curve is represented by a straight 45° diagonal line (no heterogeneity). The ‘concavity’ of the curve represents the concentration of cases through the year. To calculate and plot the Lorenz curve, the study-specific proportions were ranked by ascending order of incidence. The ranked cumulative incidence was plotted against the cumulative proportion of the year.

The Gini coefficient is a summary statistical measure of inequality with values ranging from 0 (absolute equality) to 1 (absolute inequality). For this study, a higher Gini coefficient indicated a more uneven distribution of cases through the year. With the same ranked data used to plot the Lorenz curve, Gini Coefficients, G, were calculated for each study using the formula presented by Lee [43]. To take into account variation in each individual study, using the program R (v. 2.12.2) (RDC 2004), a Gini Coefficient and its error structure was estimated for each study. For each disease, an overall Gini Coefficient and associated variance was estimated by bootstrapping [44]. These measures have previously been extensively applied to demonstrate socio-economic inequality, and more recently in infectious disease epidemiology to assess seasonality [45] and allow easily interpretable comparisons across diseases [46].

### Monthly Seasonality Index

For each disease, a monthly seasonality index [40] was applied to describe both overall and regional variations in seasonal patterns. To create overall monthly seasonality indices for each disease, the study-specific monthly proportions were averaged and converted to percentages. For example, if March had a value of 173, it would mean that the proportion of cases in March was 73% higher than the 12 month average. A plotted confidence interval of plus and minus one standard deviation accompanied each overall monthly index.

To obtain region specific monthly indices, the study-specific monthly proportions from each region were averaged and plotted. The strength of this index is that this method reduces the variability that may occur from combining data from years with high number of cases to years with low annual numbers, because it focuses on the relative monthly incidence [40]. The index allows a visual comparison of monthly seasonal patterns across diseases and among regions. It also describes where (region) seasonality is evident and when (months) incidence is highest.

## Results

### Systematic Review

**Figure 1:**
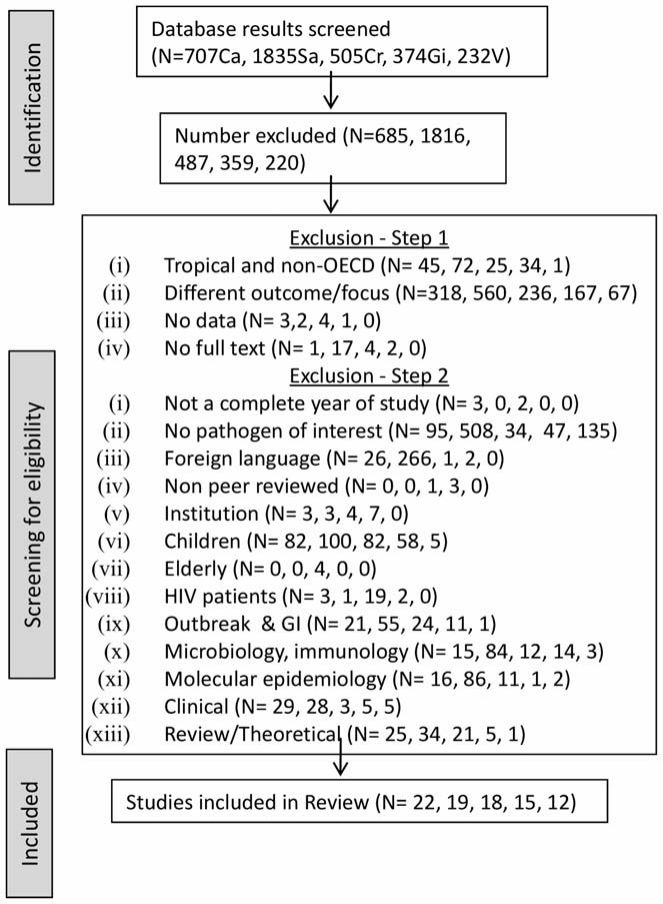
Flow chart illustrating criteria for study selection. The grey boxes represent the three major steps relating to article selection process. N is the total number of papers found across all three database searches; the suffixes represent the disease (Ca-Campylobacteriosis, Sa-Salmonellosis, V-VTEC, Cr-Cryptosporidiosis, Gi-Giardiasis). This order is maintained throughout the diagram.

The results of the search strategy and study selection for each of the five pathogens are given in Figure 1. Across all the diseases, a total of 3652 titles and abstracts were screened for eligibility. Of these, 86 studies from 19 countries conducted across 1960–2010 met the selection criteria and contributed to the systematic review and subsequent analyses (Table S1).

### Seasonality

#### Lorenz curve and Gini coefficient

**Figure 2:**
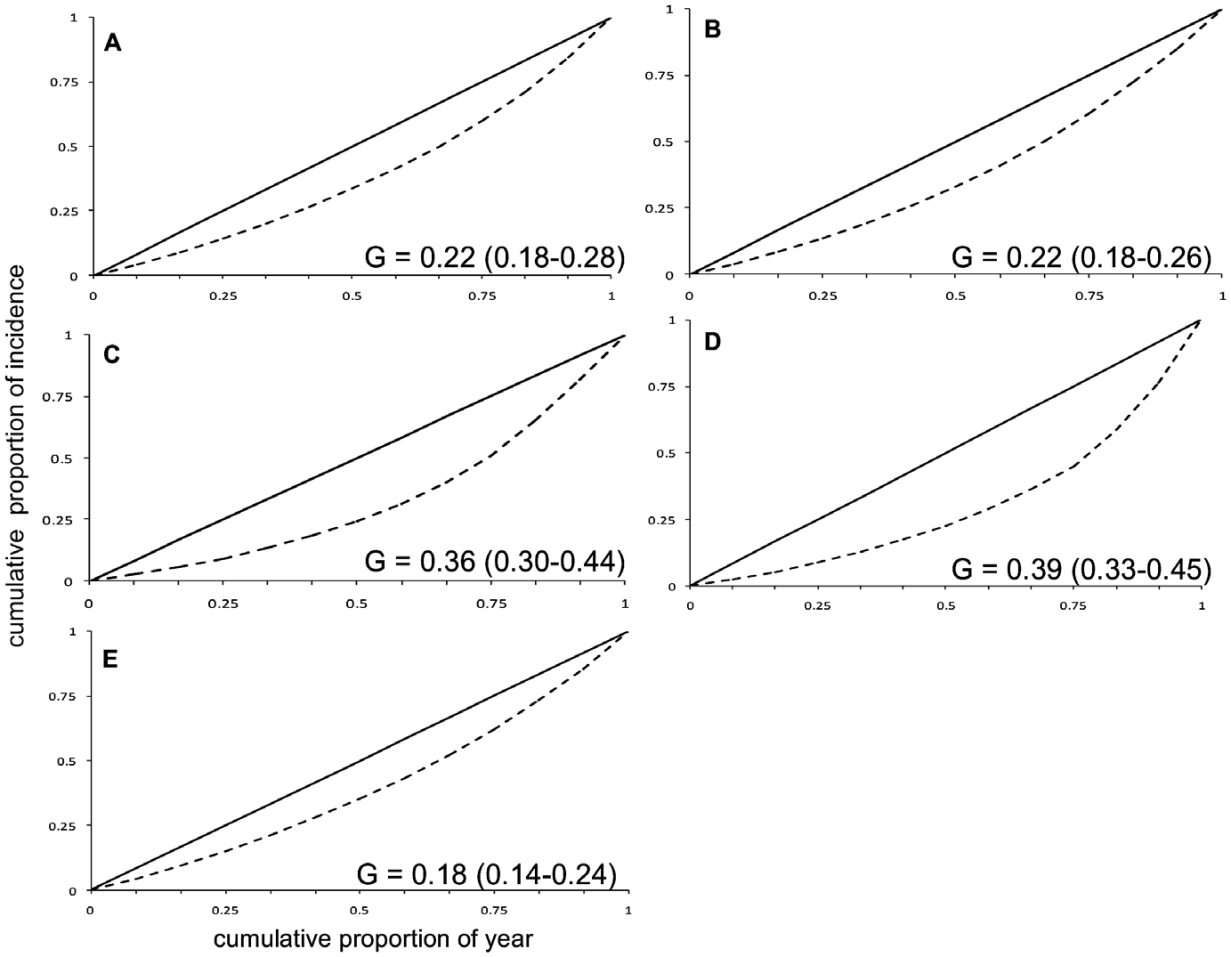
The Lorenz curves and corresponding Gini co-efficient for each disease. Disease curves showing cumulative distribution of disease incidence as a function of proportion of the year. The solid black line represents equal incidence through the year, and the dotted line represents the cumulative incidence. (A-campylobacteriosis, B-salmonellosis, C-VTEC, D-cryptosporidiosis, E-giardiasis).

For all diseases, the Lorenz curve and corresponding Gini coefficient indicated the departure from uniformly distributed incidence through the year. The Lorenz curve for campylobacteriosis, salmonellosis and giardiasis was even with the giardiasis curve being a little shallower. The Lorenz curve for VTEC indicated gradual but distinct temporal variations. Cryptosporidium showed a sharp skew in the curve indicating a clear, narrow peak in temporal incidence. The corresponding Gini coefficients were as follows: campylobacteriosis 0.22 (0.18–0.28), salmonellosis 0.22 (0.18–0.26), VTEC 0.36 (0.30–0.44), cryptosporidiosis 0.39 (0.33–0.45), giardiasis 0.18 (0.14–0.24) (Figure 2).

#### Monthly seasonality index

**Figure 3:**
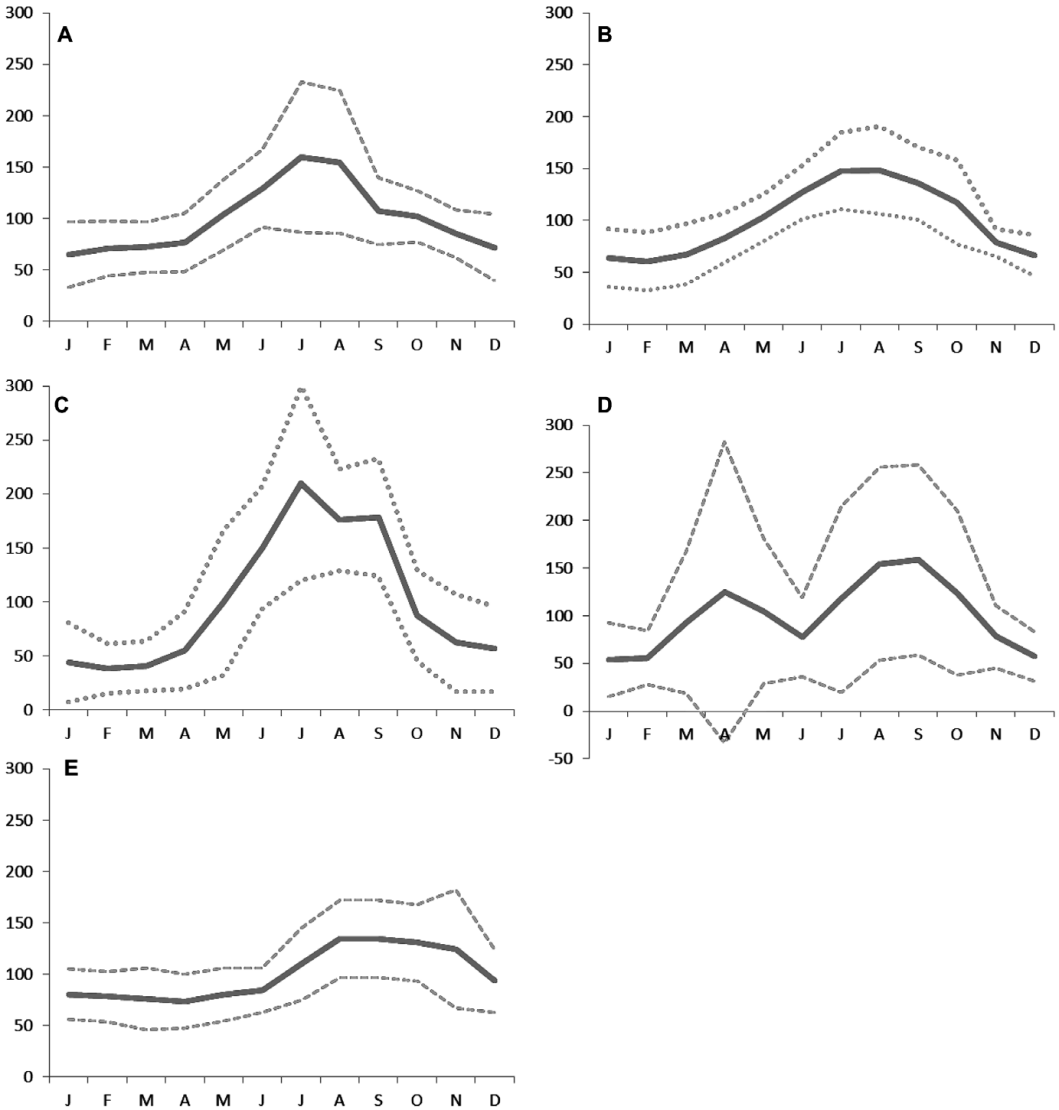
Disease specific overall monthly seasonality indices with plotted confidence intervals. Months and seasons shown refers to month/seasons of the northern hemisphere (i.e. January = month 1) and adjusted by six months for the southern hemisphere (i.e. January = month 7). Seasons are December, January, February (winter), March, April, May (spring), June, July, August (summer), September, October, November (autumn). CI of plus and minus one standard deviation is plotted. (A-campylobacteriosis, B-salmonellosis, C-VTEC, D-cryptosporidiosis, E-giardiasis).

When disease incidence was pooled up to the multi-national scale, seasonal patterns were distinct with four of the five diseases showing clear peaks. Regional variations in these patterns were evident for all the diseases (Figure 3).

#### Campylobacteriosis

**Figure 4:**
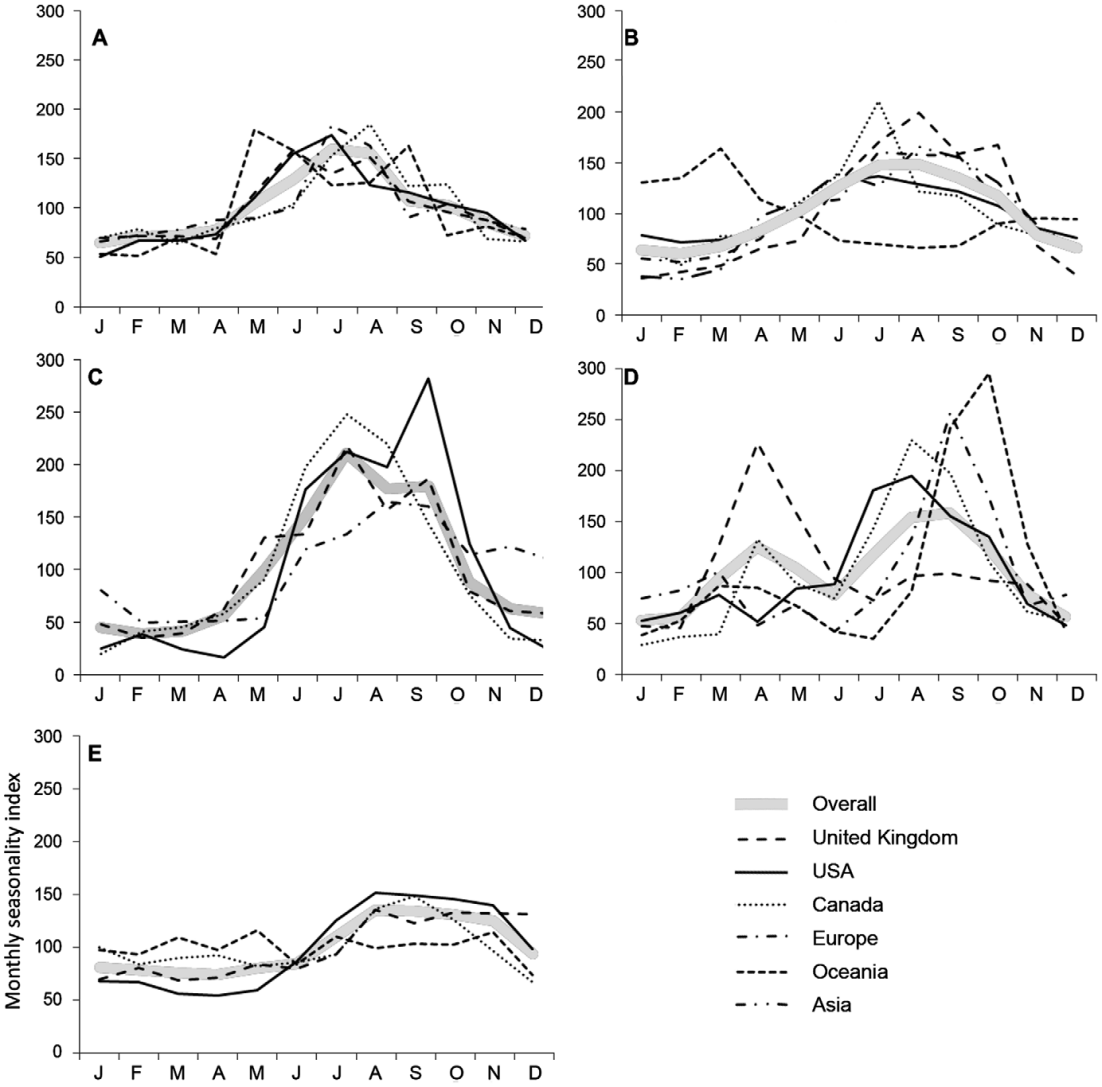
Disease specific, regional monthly seasonality indices. Months and seasons shown refers to month/seasons of the northern hemisphere (i.e. January = month 1) and adjusted by six months for the southern hemisphere (i.e. January = month 7). Seasons are December, January, February (winter), March, April, May (spring), June, July, August (summer), September, October, November (autumn). CI of plus and minus one standard deviation is plotted. (A-campylobacteriosis, B-salmonellosis, C-VTEC, D-cryptosporidiosis, E-giardiasis).

The overall monthly seasonality index for campylobacteriosis peaked in summer ( July-August) (Figure 3a). Regionally, North America, UK, Europe and Canada peaked between June-August with the UK peaking the earliest in June and Canada the latest in August. When adjusted for northern hemisphere seasons, the Oceanic region showed two seasonal peaks, once in May and again in September (Figure 4a).

#### Salmonellosis

The overall monthly seasonality index curve for salmonellosis was wider, spreading from summer-early autumn ( June-September) (Figure 3b). US peaked first in June-July followed by Canada and Europe in July, and then the UK and Asia in August (Figure 4b). The disease incidence in these countries exhibited a decrease through the rest of the year. Adjusted for the northern hemisphere season, the Oceanic region showed a peak in spring (February-March) and a relatively small increase over autumn (October-November).

#### VTEC

The overall index for VTEC indicated a dominant peak in summer ( July) with a lesser peak in September (Figure 3c). The UK and Canada peaked first in July and Europe in August (Figure 4c). US also showed a slight peak in summer ( July), but a dominant peak in autumn (September). While North America, Canada and the UK showed a decrease in winter, Europe remained above the overall average for autumn-winter (October-January). 

#### Cryptosporidiosis

The overall monthly index for cryptosporidium indicated a clear bi-modal peak, in spring (April) and late summer-early autumn (August-September), with the latter being considerably larger (Figure 3d). Separately, all countries considered here had one major peak (Figure 4d). The adjusted incidence in Oceanic countries (Australia, New Zealand) together with the UK exhibited a strong spring peak (April) with an additional second smaller autumn peak (September) shown by the Oceanic countries. North America, Canada, and the rest of Europe displayed a late summer peak, with Canada showing a small spring peak. In all countries represented, the lowest number of cases was recorded in winter.

#### Giardiasis

The monthly seasonality index for giardia showed a small summer peak (Figure 3e). Here, Oceania, Europe and Canada displayed a small spring peak (March-May), while the US, UK and Canada showed a larger summer peak ( July-September) (Figure 4e). The UK and Canada peaked first around July, followed by US in August and Europe in September.

## Discussion

Based on data from 86 studies, regular, cyclical patterns were observed for all enteric zoonotic diseases, with campylobacteriosis, salmonellosis, VTEC and cryptosporidiosis displaying distinct season associated peaks. This review used easily interpretable measures of seasonality across diseases to compare monthly incidence patterns among major regions. Although global reviews of general seasonality in these diseases have typically focussed on the climate-disease association [10], [47] and our results are not strictly comparable with these studies, they are largely consistent with literature published on individual diseases.

There are several potential mechanisms proposed to explain seasonal patterns in enteric diseases. Although attributing causal mechanisms for seasonal patterns is beyond the scope of this study, our discussion of seasonality in these diseases is centred on two principal mechanisms believed to significantly influence disease patterns globally: (i) environmental influences on pathogen occurrence and pathogen-host interactions and (ii) population behaviour and characteristics.

### Environmental Influences on Disease Incidence

Seasons in temperate countries are typically characterised by changing weather conditions and for convenience, defined by calendar year [48]. The bacterial diseases, campylobacteriosis, salmonellosis and VTEC, showed temporal variations in incidence, indicated by the Gini index and a similar spread of seasonal peaks in summer for most regions, shown by the seasonality index. Bacterial pathogens are sensitive to changes in heat, moisture, oxygen, light and nutrients [49]. Increased temperatures could enhance pathogen survival and proliferation [9], [50], potentially increase pathogen load in animal reservoirs [30] and prolong transmission seasons [51]. The consistent summer peak in bacterial diseases suggests the direct effect of a large-scale environmental influence on a shared exposure route. As food is the dominant vehicle for transmission of these pathogens in many countries [52], [53], [54], it is probable that higher temperatures associated with summer increase the risk of food borne transmission [50]. For example, a study undertaken across six European countries reported a strong association between average monthly temperature and campylobacter incidence in both broiler chickens and humans [55]. Higher campylobacter prevalence in flocks and retail meat in summer have also been shown [56]. Summer peaks in human VTEC infection have been associated with ground beef consumption [57] with similar findings for other bacterial pathogens [58], [59]. For food borne illnesses, sustained warmer temperatures could increase length of transmission seasons, enhancing opportunities for food handling errors leading to seasonal enteric disease outbreaks [60]. This premise is supported by studies conducted in New Zealand [61] and the United Kingdom [62] where the association of bacterial enteric disease with climatic variables has weakened over time, suggesting that targeted health policies and obligatory industrial regulations were effective. This focussed control of known transmission mechanisms may explain why the Gini indices for campylobacteriosis and salmonellosis were smaller than for VTEC, which is relatively less common and could have different dominant reservoirs and transmission pathways (discussed below). Therefore, although environmental influences can directly limit disease establishment and transmission [57], [58] or conversely, promote disease spread and severity [59], there is evidence that a proactive multidisciplinary approach to disease control and prevention could mitigate some of these impacts, particularly for predominantly food borne pathogens.

Changes in ambient physical conditions can also influence environmentally mediated, pathogen transmission pathways, playing an important role in driving seasonality in these diseases [37], [63]. For example, summer highs of campylobacteriosis in temperate countries have been hypothesised to occur as a result of summer increases in house fly density [64], with flies acting as mechanical vectors of transmission, thereby enhancing seasonal prevalence [65], [66].

Spring peaks in cryptosporidiosis incidence may be related to contamination of water supplies through heavy rainfall events. A study in North West England showed that in areas with marked seasonal patterns, cryptosporidiosis was associated with increased rainfall [67]. In the same geographic region, a significant association between maximum river flows and cryptosporidiosis cases in spring were found, suggesting that increased seasonal pathogen load coupled with heavy rainfall could result in seasonally high disease rates [68]. Unusually heavy runoff from spring snow melt has also been implicated in protozoan disease outbreaks [69]. For water borne diseases such as cryptosporidiosis, quantifying pathogen loads in water sources preceding and subsequent to heavy rainfall events could broaden our understanding of environmentally moderated seasonal drivers.

Seasonal land use patterns that increase contact between animal reservoirs and human populations may be key determinants of regional and temporal differences in seasonality. For cryptosporidiosis, all represented regions had a prominent seasonal peak, commonly with a second smaller peak (bi-modal seasonal pattern), suggesting important regional variations in disease drivers. For example, both New Zealand and the UK displayed a prominent spring peak along with a smaller one in autumn for oceanic countries. Young livestock are a significant reservoir of enteric pathogens [70], [71] and synchronisation of agricultural practices such as calving take place in both New Zealand and the UK in spring [72]. In New Zealand the majority of human cases in spring have been attributed to the bovine strain with peaks in autumn due to the human strain [73], [74]. Analogously, the UK has reported similar seasonal shifts in cryptosporidium strains affecting humans, with zoonotic sources hypothesised to dominate in spring and anthroponotic sources in autumn [75], [76].

Comparable evidence exists for other enteric pathogens, highlighting the importance of land use patterns in driving strain specific, seasonal disease incidence [77]. For example, the oceanic countries also showed spring peaks in campylobacteriosis and salmonellosis. In New Zealand, campylobacteriosis source attribution studies have found that ruminant strains pose a greater risk to rural children [78]. Also, peak infection rates of a predominantly livestock strain of salmonella in humans is reported to track farming practices [79]. Unfortunately, due to lack of published data on the monthly incidence of specific strains for all the pathogens considered here, we were unable to address this level in our review.

The comparatively high Gini index and narrow summer peak for VTEC indicate a seasonally restricted exposure, which could be related to seasonal agricultural activities. Agricultural variables such as cattle density [80], farm density [81], animal manure applied to soil [19], and farm visits [82] have been recognised as important risk factors for VTEC infection. Moreover, livestock are a major reservoir, with seasonal patterns of pathogen shedding, generally increasing in the warmer months [83]. Also, as young stock have higher prevalence [84], the seasonality of activities such as calving could also contribute to seasonal patterns in infections. The high index could indicate seasonally high incidences due to difficulties associated with identifying, quantifying and controlling environmental exposures. Clarifying the role of land use activities in driving seasonal patterns of VTEC infection would be useful to determine the extent of zoonotic transmission of this disease.

### Population Influences on Disease Incidence

Periodic oscillations in host characteristics are also an important seasonal forcing mechanism driving epidemiological patterns in enteric diseases [15]. Seasonal, host related factors could be cultural or socio-economic [85], [86], linked to host lifestyle [87] or cyclical immunity [88]. For example, foreign travel plays an important role in explaining summer peaks in campylobacteriosis and salmonellosis in the UK and Europe, with regional differences in seasonality attributed to countries visited and dominant strains prevalent in these destinations [21], [89], [90]. As most studies included here did not distinguish between endemic and travel cases, we were unable to quantify seasonal differences among the two groups [30].

Our results for giardiasis indicated minimal inequality in incidence through the year as suggested by the Gini coefficient and seasonality index. Giardiasis incidence and spread is thought to be mainly a result of anthroponotic risk factors and transmission [91], [92], [93], [94]. So, the slightly elevated summer highs shown in the USA, UK and Canada may imply increased person-to-person transmission in warmer months as a consequence of outdoor activities [95] and exposure to untreated water sources [96]. Similarly, summer peaks in cryptosporidiosis incidence in USA and Canada as shown here could be a consequence of host related factors such as recreational water use [97] and seasonal contact with livestock [98]. As recent studies have found that social connectedness is an important predictor of diarrhoeal disease incidence [99], quantifying the relevance of specific population behaviours to seasonality in these diseases would be useful.

Although few studies have documented interactions between environmental and population factors, these effects are not mutually exclusive. For example, although a study assessing the relationship between climate variables, *Salmonella* incidence in retail chicken and human cases in Canada found a positive association with temperature, it was summer barbequing and gardening that were identified as primary risk factors [30]. In New Zealand, population drinking water quality was found to modify the positive association between rainfall and cryptosporidiosis, with better quality drinking water having a protective effect [100].

Environmental effects are expected to have a considerable impact on existing patterns in human geography in the future [101], in some instances modifying environment-pathogen-host interactions substantially. As temperate, developed countries are expected to experience increasing climate variability [102] land use intensification and urbanisation [103], with respect to enteric zoonotic diseases in particular, efforts to reconcile human health with environmental and population linked change present a major challenge. Evaluating seasonality provides a baseline pattern of the complex interplay of climatic and non-climatic seasonally mediated factors acting at different spatio-temporal scales, and interacting along a continuum of influence. Targeted ecological studies combined with microbiological analysis and population intensive monitoring would be useful in elucidating the relative importance of specific enteric pathogens (and strains), their transmission, spread and seasonal significance on enteric disease burden.

This review highlights the regionally and seasonally varying, context dependant, inter-relationships between environmental processes, pathogen physiology and intrinsic host attributes as drivers of enteric disease patterns. Nonetheless, our study has certain shortcomings. First, as we aimed to assess seasonal patterns in zoonotic diseases in the general population, we did not focus on host characteristics and seasonal patterns for particular demographic groups (such as children, the elderly or immune suppressed), possibly resulting in a simplification of seasonal patterns (e.g. [104]). Second, as a consequence of the keywords used and our concentration on studies conducted in English, it is possible that our search strategy missed some relevant studies. However, by searching three different databases and checking reference lists of all relevant articles and reviews, we are confident we identified the majority of the appropriate literature. Third, as our primary aim was to describe seasonal patterns, we included studies using different designs and methodologies (e.g. [47]), assuming differences would be independent of seasonal trends over time [26], [105].

The Lorenz curve and Gini index are influenced by sample variability and length of study, and high values may not be indicative of actual seasonality. Although data were standardised to a monthly scale, it is accepted that the studies being of varying duration could affect interpretation of results. The current approach was validated by excluding studies conducted for a year and since no significant change was found, they were included in the final review. While recognising the limitations of this method, when taken together with the seasonality index and the seasonal patterns documented by the individual authors (see Table S1), the Gini index remains useful as a relative indicator of potentially seasonally associated variation and warrants further research. Combining data from geographically and climatically diverse areas implies that subtle differences in seasonal patterns among countries may have been lost, although we attempted to reduce this effect by selecting studies from temperate areas only. Finally, the monthly seasonality index was biased towards countries with presumably more established research settings [106]. However, regional grouping highlighted potential gaps in the availability of comparable, published data (e.g. salmonellosis in New Zealand) [107]. The objective of this study was to evaluate whether enteric zoonotic diseases exhibited patterns coincident with season and to review our results in the light of two primary mechanisms thought to influence global enteric disease patterns. It was assumed that if the studies were reasonably representative of the general population, study duration was sufficient, and reporting systems and target populations did not vary greatly between years, existing seasonal patterns would be cyclical and distinguishable.

### Conclusions

Seasonality in enteric diseases with predominantly animal reservoirs is ubiquitous. The diseases reviewed show consistent seasonal patterns across trans-national boundaries, albeit with regional variations in these patterns. Understanding disease specific seasonal patterns is important for improving existing disease surveillance methods, generating appropriate prevention strategies, developing valid prediction models, and enhancing cross-border cooperation. Although an individual level focus is often necessary to infer causality [108], assessing population level patterns allows environmental processes, which typically function at international scales, to be integrated into the public health framework [35]. This perspective is particularly important for those diseases where trans-boundary environmental change plays a pivotal role in disease incidence [109], which may not be measurable at the individual scale. Regional variations in seasonal patterns provide an insight into the complex hierarchical and nonlinear nature of interactions between environmental, pathogen and host specific factors and transmission opportunities. These finer scale patterns also highlight regions that follow the expected pattern and those that are outliers, which is crucial information to establish region specific determinants of disease variability. Results from this review encourage targeted ecological, microbiological and population focused investigations to uncover causal mechanisms driving seasonality, particularly at the regional level. This need is particularly important as this review indicates that local landscape changes and population dynamics, together with larger scale longer-term climate change, could have far-reaching direct and indirect consequences for future enteric disease risk.

## Supporting Information

Table S1 **Attributes of 86 studies across five enteric diseases selected for review.**
(PDF)
