# Supplementary material for: Seasonality in Human Zoonotic Enteric Diseases: A Systematic Review
Source: PLoS One. 2012 Apr 2;7(4):e31883. doi: 10.1371/journal.pone.0031883 (PMC3317665; doi:10.1371/journal.pone.0031883)
Supplement: Table S1 — Attributes of 86 studies across five enteric diseases selected for review. (PDF) [file pone.0031883.s001.pdf]

Supplemental Material

Manuscript title: Seasonality in human enteric diseases: A Systematic Review

Names of the authors: Aparna Lal[1], Simon Hales[1], Nigel French[2], Michael Baker[1]

Supplemental Material, Table 1. Attributes of 86 studies across five enteric diseases selected for review

Study Citation	Location, Years of Study	Study Design, Sample Size	Temporal Scale	Age Range (Yrs)	Peak season noted by the authors											
					J	F	M	A	M	J	J	A	S	O	N	D
		**(A) CAMPYLOBACTERIOSIS**														
**USA**																
Hopkins and Olmsted 1985	Colorado, 1981-1982	Surveillance, 1185	Monthly	0-≥60								+	+			
Blaser et al. 1982	Denver, 1978-1980	Surveillance, 124	Monthly	0->60							+	+				
Altekruse et al. 1999	US, 1996	Surveillance (FoodNet)[a]	Monthly	0-≥60						+	+	+				
**CANADA**																
Thompson et al. 1986	Ontario, 1978-1985	Surveillance, 412	Monthly	0->60						+	+	+	+			
Michaud et al. 2004	Quebec, 2000-2001	Prospective Matched Case-Control, 158 (cases), 314 (controls)	Monthly	0->60							+	+	+			
Allard et al. 2010	Montreal, 1990-2006	Surveillance[a]	Weekly	None[b]												
**UNITED KINGDOM**																
Kendall and Tanner 1982	UK, 1979-1981	Surveillance, 518	Monthly	0->60						+	+	+				
Bradshaw et al. 1980	Chelmsford, 1977-1978	Surveillance, 109	Monthly	0->60						+	+	+	+			
Skirrow 1987	England, 1983-1984	Surveillance, 1873	Monthly	0->60					+	+						
Sopwith et al. 2003	NW England, 1997-1999	Surveillance, 3939	Monthly	0->60					+	+						
Jones et al. 1990	Lancaster, 1988-1989	Surveillance[a]	Monthly						+	+						
Sibbald and Sharp 1985	Scotland, 1978-1982	Surveillance, 7808	Monthly	0->60					+	+						
**EUROPE**																
Jepsen et al. 2009	Funen, Denmark, 1995-2004	Surveillance, 2984		None[b]												
Lassen and Kapperud 1984	Norway, 1980-1982	Surveillance, 249	Monthly	0->60							+	+				
Denis et al. 2009	Brittany, France, 2003-2004	Surveillance, 177	Monthly	0->60							+	+	+			
Samonis et al. 1997	Crete, Greece, 1992-1994	Surveillance, 169	Monthly	0->15							+	+				+
Studahl and Anderrson 2000	Älvsborg, Sweden,1995	Retrospective Matched Case-Control, 101 (cases), 198 (controls)	Monthly	0->60								+	+	+		
Nakari et al. 2010	Finland, 1996-2001	Surveillance, 5650	Monthly	0->60						+	+	+	+			
Karelampi et al. 2007	Helsinki, Finland, 1996, 2002,2003	Surveillance, 307	Monthly	19->60							+	+	+			

					Peak season noted by the authors											
**Study Citation**	**Location, Years of Study**	**Study Design, Sample Size**	**Temporal Scale**	**Age Range (Yrs)**	**J**	**F**	**M**	**A**	**M**	**J**	**J**	**A**	**S**	**O**	**N**	**D**
Liesenfield et al. 1993	Germany, 1989-1991	Surveillance, 98	Quarterly,	0->60				+	+	+						
Cabrita et al. 1992	Portugal, 1984-1989	Surveillance, 150	Monthly	0->19	+	+										
**OCEANIA**																
Brieseman et al. 1985	Christchurch, New Zealand, 1981-1983	Surveillance, 517	Monthly	0->60	+	+									+	+
		**(B) SALMONELLOSIS**														
**USA**																
Arshad et al. 2007	Michigan, 1995-2001	Surveillance, 6797	Monthly	0-≥60						+	+					
Oloya et al. 2007	Dakota, 2000-2005	Surveillance, 286	Monthly	None[b]						+	+	+				
Altekruse et al. 1999	US, 1996	Surveillance (FoodNet)[a]	Monthly	0-≥60						+	+	+				
Cherubin et al. 1996	New York, 1965-1966	Surveillance, 2200	Monthly	0->60							+	+	+			
Aserkoff et al. 1970	US, 1963-1967	Surveillance, 20,000	Monthly	0->60							+	+	+	+		
Younus et al. 2006	Michigan, 1995-2001	Surveillance, 1296	Monthly	0->60					+	+	+					
**CANADA**																
Ravel et al. 2010	Ontario2005-2008	Surveillance (C-EnterNet), 216	Monthly	0-≥60						+	+					
**UNITED KINGDOM**																
Banatvala et al. 1999	North East Thames, 1993	Surveillance, 1760	Monthly	0->60						+	+	+				
Sharp and Heyman 1976	Scotland, 1967-1974	Surveillance, 194	Monthly	0->60								+	+	+		
Skirrow 1987	England, 1983-1984	Surveillance, 1149	Monthly	0->60							+	+				
**EUROPE**																
Leisenfield et al. 1993	Germany, 1989-1991	Surveillance, 264	Monthly	0->60							+	+	+			
Samonis et al. 1997	Crete, Greece, 1992-1994	Surveillance, 491	Monthly	0-≥15						+	+	+				
Cabrita et al. 1992	Portugal, 1984-1989	Surveillance, 416	Monthly	0-≥19							+	+	+			
Collard et al. 2008	Belgium, 1970-2005	Surveillance[a]	Monthly								+	+	+			
**OCEANIA**																
D'Souza et al. 2004	Australia, 1991-2001	Surveillance[a]	Monthly	None[b]	+	+	+									
**ASIA**																

Study Citation	Location, Years of Study	Study Design, Sample Size	Temporal Scale	Age Range (Yrs)	Peak season noted by the authors											
					J	F	M	A	M	J	J	A	S	O	N	D
Cho et al. 2006	Republic of Korea, 2003	Surveillance, 376	Monthly	0->60						+	+	+	+			
Cho et al. 2008	Republic of Korea, 2004-2006	Surveillance, 957	Monthly	0->60							+	+	+			
Toyofuku 2008	Japan, 1998-2004	Surveillance[a]	Monthly	None[b]							+	+	+			
**(C) VTEC**																
**USA**																
Altekruse et al. 1999	US, 1996	Surveillance[a]	Monthly	0-≥60									+	+	+	
**CANADA**																
Pai et al. 1988	Alberta, 1984-1986	Surveillance, 166	Monthly	0->60									+	+	+	
Waters et al. 1994	Alberta, 1987-1991	Surveillance, 1450	Monthly	0->60								+	+	+		
Michel et al. 1999	Ontario, 1990-1995	Surveillance, 3001	Monthly	None[b]								+				
**UNITED KINGDOM**																
Thomas et al. 1996	England and Wales, 1992-1994	Surveillance, 1468	Monthly	0->60									+	+	+	
Willshaw et al. 2001	England and Wales, 1995-1998	Surveillance, 3429	Monthly	0->60									+	+	+	
Chapman et al. 1989	Sheffield, England, 1986-1987	Prospective Matched Case-Control, 36 (cases), 229 (controls)	Monthly	4->60									+	+		
Money et al. 2010	England, Wales, Scotland, 2000-2006	Surveillance, 9507	Monthly	None[b]									+	+	+	
MacDonald et al. 1996	Grampian Region, Scotland, 1988-1990	Surveillance, 95	Monthly	0->60								+	+	+	+	
Waters et al. 1994	Scotland, 1987-1991	Surveillance, 505	Monthly	0->60									+	+	+	
Carroll et al. 2005	Ireland, 2002-2004	Surveillance, 207	Monthly										+	+	+	
**EUROPE**																
Eklund et al. 2005	Finland, 1998-2002	Surveillance, 124	Monthly	None[b]									+	+	+	
Kistemann et al. 2004	Sweden, 1995-1999	Surveillance, 525	Monthly	0->60											+	+

					Peak season noted by the authors											
**Study Citation**	**Location, Years of Study**	**Study Design, Sample Size**	**Temporal Scale**	**Age Range (Yrs)**	**J**	**F**	**M**	**A**	**M**	**J**	**J**	**A**	**S**	**O**	**N**	**D**
		**(D) CRYPTOSPORIDIOSIS**														
**USA**																
Naumova et al. 2000	Massachusetts, 1993-1996	Surveillance, 230	Daily	0->60								+	+	+		
Dietz et al. 2000a	7 States, 1997-1998	Surveillance (FoodNet), 1023	Monthly	0->60							+	+	+	+		
Dietz et al. 2000b	47 States, 1995-1998	Surveillance, 11612	Monthly	0->60							+	+	+	+		
Roy et al. 2004	7 States, 1999-2001	Retrospective Matched Case-Control, 282 (cases) 490 (controls)	Monthly	0->60						+	+	+	+			
Amin 2002	48 States, 2000	Surveillance, 121	Monthly	0->60			+							+		
**CANADA**																
Pintar et al. 2009	Ontario, 2005-2007	Case Control,36 (cases) 803 (controls)	Monthly	0≥60							+	+	+			
Majowicz et al. 2001	Ontario, 1996-1997	Surveillance, 451	Quarterly	0≥60								+	+	+		
Laupland and Church 2005	Calgary, 1999-2002	Surveillance, 173	Monthly	0->60	no seasonality noted											
Mann et al. 1986	Manitoba, 1983-1984	Surveillance, 39	Monthly	0->60								+	+			
**UNITED KINGDOM**																
Callaghan et al. 2009	West Ireland, 2004-2007	Surveillance, 569	Monthly	None[b]			+	+	+							
Garvey and McKeown 2009	Ireland, 2004-2006	Surveillance, 1363	Monthly	0->60				+	+	+						
Zintl et al. 2009	Ireland, 2000-2007	Surveillance, 199	Monthly	0->60			+	+	+							
Sopwith et al. 2005	North West England, 1996-2000	Surveillance, 3711	Weekly	0≥45				+	+	+						
Chalmers et al. 2009	England and Wales, 2000-2003	Surveillance, 7829	Monthly	0->60								+	+	+		
Pollock et al. 2009	Scotland, 2005-2007	Surveillance, 560	Monthly	0->60								+	+	+		
**EUROPE**																
Derouin et al. 2010	France, 2006-2009	Surveillance (Anofel), 407	Monthly	0->60								+	+	+		
Wielinga et al. 2008	Netherlands, 2003-2005	Surveillance, 91	Monthly	0->60									+	+		

Study Citation	Location, Years of Study	Study Design, Sample Size	Temporal Scale	Age Range (Yrs)	Peak season noted by the authors											
					J	F	M	A	M	J	J	A	S	O	N	D
**OCEANIA**																
Snel et al. 2009	New Zealand, 1997-2006	Surveillance, 8212	Monthly	0->60									+	+	+	
**(E) GIARDIASIS**																
**USA**																
Dennis et al. 1993	New Hampshire, 1984	Retrospective Case-Control, 273 (cases), 375 (controls)	Monthly	0≥55							+	+	+	+		
Birkhead and Vogt 1989	Vermont, 1983-1986	Surveillance, 1211	Monthly	0->60							+	+	+	+	+	
Naumova et al. 2000	Massachusetts, 1993-1996	Surveillance, 4508	Monthly	0->60							+	+	+	+	+	
Wright et al. 1977	Colorado, 1972-1973	Retrospective Matched Case-Control, 256 (cases), 256 (controls)	Monthly	0≥60							+					
Addiss et al. 1992	Wisconsin, 1981-1988	Surveillance, 9836	Monthly	0->60								+	+	+		
Navin et al. 1985	Nevada, 1982-1983	Retrospective Matched Case-Control, 45 (cases), 45 (controls)	Bi-weekly	0->60										+	+	
**CANADA**																
Laupland and Church 2005	Calgary, 1999-2002	Surveillance, 552	Monthly	0->60								+	+	+	+	
Odoi et al. 2003	Ontario, 1990-1998	Surveillance, 17031	Monthly	0≥60							+	+	+			
Greig et al. 2001	Ontario, 1990-1998	Surveillance, 25289	Monthly	0≥60								+	+	+		
**UNITED KINGDOM**																
Flanagan 1992	Scotland, 1983-1989	Surveillance[a]	Monthly	0->60							+	+	+			
Breathnach et al. 2010	South West London, 1999-2005	Surveillance, 819	Monthly	0->60										+	+	+
Gray et al. 1994	Avon and Somerset, 1992-1993	Matched Case- Control, 74 (cases), 108 (controls)	Monthly	0->60								+	+	+	+	
**OCEANIA**																
Kettlewell et al. 1998	Tasmania, 1995-1997	Surveillance, 456	Monthly	0->60	no seasonality noted											
Snel et al. 2009	New Zealand , 1997-2006	Surveillance, 16471	Monthly	0->60			+	+	+							
Hoque et al. 2002	Auckland, New Zealand, 1996-2000	Surveillance, 2510	Monthly	0->60		+	+									

[a] Total sample size not provided in the article

[b] Age range of study population not provided in the article

References

Addiss DG, Davis JP, Roberts JM, Mast EE. 1992. Epidemiology of giardiasis in Wisconsin: increasing incidence of reported cases and unexplained seasonal trends. Am J Trop Med Hyg 47(1): 13-19.

Allard R, Plante C, Garnier C, Kosatsky T. 2010. The reported incidence of campylobacteriosis modelled as a function of earlier temperatures and numbers of cases, Montreal, Canada, 1990-2006. Int J Biometeorol; doi: 10.1007/s00484-010-0345-x [Online 27 July 2010].

Amin OM. 2002. Seasonal prevalence of intestinal parasites in the United States during 2000. American Journal of Tropical Medicine and Hygiene 66 (6): 799-803.

Arshad MM, Wilkins MJ, Downes FP, Rahbar MH, Erskine RJ, Boulton ML, et al. 2007. A registry-based study on the association between human salmonellosis and routinely collected parameters in Michigan, 1995-2001. Foodborne Pathog Dis 4(1): 16-25.

Aserkoff B, Schroeder SA, Brachman PS. 1970. Salmonellosis in the United States--a five-year review. Am J Epidemiol 92(1): 13-24.

Banatvala N, Cramp A, Jones IR, Feldman RA. 1999. Salmonellosis in North Thames (East), UK: Associated risk factors. Epidemiol Infect 122: 201-207.

Birkhead G, Vogt RL. 1989. Epidemiologic surveillance for endemic Giardia lamblia infection in Vermont. The roles of waterborne and person-to-person transmission. Am J Epidemiol 129(4): 762-768.

Blaser MJ, Taylor DN, Feldman RA. 1983. Epidemiology of Campylobacter jejuni infections. Epidemiol Rev 5: 157-176.

Bradshaw MJ, Brown R, Swallow JH, Rycroft JA. 1980. Campylobacter enteritis in Chelmsford. Postgrad Med J 56(652): 80-84.

Breathnach AS, McHugh TD, Butcher PD. 2010. Prevalence and clinical correlations of genetic subtypes of Giardia lamblia in an urban setting. Epidemiol Infect 138(10): 1459-1467.

Brieseman MA. 1985. The epidemiology of campylobacter infections in Christchurch 1981-83. N Z Med J 98(779): 391-393.

Cabrita J, Pires I, Vlaes L, Coignau H, Levy J, Goossens H, et al. 1992. Campylobacter-Enteritis in Portugal - Epidemiologic Features and Biological Markers. Eur J Epidemiol 8(1): 22-26.

Carroll AM, Gibson A, McNamara EB. 2005. Laboratory-based surveillance of human verocytotoxigenic Escherichia coli infection in the Republic of Ireland, 2002-2004. J Med Microbiol 54(12): 1163-1169.

Chapman PA, Wright DJ, Norman P. 1989. Verotoxin-producing Escherichia coli infections in Sheffield: cattle as a possible source. Epidemiol Infect 102(3): 439-445.

Cherubin CE, Fodor T, Denmark L, Master C, Fuerst HT, Winter J. 1969. The epidemiology of salmonellosis in New York City. Am J Epidemiol 90(2): 112-125.

Cho SH, Kim JH, Kim JC, Shin HH, Kang YH, Lee BK. 2006. Surveillance of bacterial pathogens associated with acute diarrheal disease in the Republic of Korea during one year, 2003. J Microbiol 44(3): 327-335.

Cho SH, Shin HH, Choi YH, Park MS, Lee BK. 2008. Enteric bacteria isolated from acute diarrheal patients in the Republic of Korea between the year 2004 and 2006. J Microbiol 46(3): 325-330.

Collard JM, Bertrand S, Dierick K, Godard C, Wildemauwe C, Vermeersch K, et al. 2008. Drastic decrease of Salmonella Enteritidis isolated from humans in Belgium in 2005, shift in phage types and influence on foodborne outbreaks. Epidemiol Infect 136(6): 771.

Denis M, Chidaine B, Laisney MJ, Kempf I, Rivoal K, Megraud F, et al. 2009. Comparison of genetic profiles of Campylobacter strains isolated from poultry, pig and Campylobacter human infections in Brittany, France. Pathol Biol (Paris) 57(1): 23-29.

Dennis DT, Smith RP, Welch JJ, Chute CG, Anderson B, Herndon JL, et al. 1993. Endemic giardiasis in New Hampshire: a case-control study of environmental risks. J Infect Dis 167(6): 1391-1395.

Derouin F, Dutoit E, de Monbrison F, Guyot K, Accoceberry I, Agnamey P, et al. 2010. Laboratory-based surveillance for Cryptosporidium in France, 2006-2009. Eurosurveillance 15(33). Available online: http://www.eurosurveillance.org/

Dietz V, Vugia D, Nelson R, Wicklund J, Nadle J, McCombs KG, et al. 2000a. Active, multisite, laboratory-based surveillance for Cryptosporidium parvum. American Journal of Tropical Medicine and Hygiene 62(3): 368-372.

Dietz VJ, Roberts JM. 2000b. National surveillance for infection with Cryptosporidium parvum, 1995-1998: What have we learned? Public Health Reports 115 (4): 358-363.

Eklund M, Nuorti JP, Ruutu P, Siitonen A. 2005. Shigatoxigenic Escherichia coli (STEC) infections in Finland during 1998-2002: a population-based surveillance study. Epidemiol Infect 133(5): 845-852.

Flanagan PA. 1992. Giardia: diagnosis, clinical course and epidemiology: a review. Epidemiol Infect 109(1): 1-22.

Garvey P, McKeown P. 2009. Epidemiology of human cryptosporidiosis in Ireland, 2004-2006: analysis of national notification data. Euro surveillance 14(8):pii 19128. Available online: http://www.eurosurveillance.org/

Gray SF, Gunnell DJ, Peters TJ. 1994. Risk factors for giardiasis: a case-control study in Avon and Somerset. Epidemiol Infect 113(1): 95-102.

Greig JD, Michel P, Wilson JB, Lammerding AM, Majowicz SE, Stratton J, et al. 2001. A descriptive analysis of giardiasis cases reported in Ontario, 1990-1998. Can J Public Health 92(5): 361-365.

Hopkins RS, Olmsted RN. 1985. Campylobacter jejuni infection in Colorado: unexplained excess of cases in males. Public Health Rep 100(3): 333-336.

Hoque ME, Hope VT, Scragg R. 2002. Giardia infection in Auckland and New Zealand: trends and international comparison. N Z Med J 115(1150): 121-123.

Jepsen MR, Simonsen J, Ethelberg S. 2009. Spatio-temporal cluster analysis of the incidence of Campylobacter cases and patients with general diarrhea in a Danish county, 1995-2004. Int J Health Geogr 8: 11; doi:10.1186/1476-072X-8-11 [Online 20 February 2009].

Jones K, Betaieb M, Telford DR. 1990. Correlation between environmental monitoring of thermophilic campylobacters in sewage effluent and the incidence of Campylobacter infection in the community. J Appl Bacteriol 69(2): 235-240.

Karenlampi R, Rautelin H, Schonberg-Norio D, Paulin L, Hanninen ML. 2007. Longitudinal study of Finnish Campylobacter jejuni and C-coli isolates from humans, using multilocus sequence typing, including comparison with epidemiological data and isolates from poultry and cattle. Appl Environ Microb 73(1): 148-155.

Kendall EJ, Tanner EI. 1982. Campylobacter enteritis in general practice. J Hyg (Lond) 88(2): 155-163.

Kettlewell JS, Bettiol SS, Davies N, Milstein T, Goldsmid JM. 1998. Epidemiology of giardiasis in Tasmania: a potential risk to residents and visitors. J Travel Med 5(3): 127-130.

Kistemann T, Zimmer S, Vagsholm I, Andersson Y. 2004. GIS-supported investigation of human EHEC and cattle VTEC O157 infections in Sweden: geographical distribution, spatial variation and possible risk factors. Epidemiol Infect 132(3): 495-505.

Lassen J, Kapperud G. 1984. Epidemiological aspects of enteritis due to Campylobacter spp. in Norway. J Clin Microbiol 19(2): 153-156.

Laupland KB, Church DL. 2005. Population-based laboratory surveillance for Giardia sp. and Cryptosporidium sp. infections in a large Canadian health region. BMC Infectious Diseases 5(72); doi:10.1186/1471-2334-5-72 [Online 16 September 2005].

Liesenfeld O, Weinke T, Hahn H. 1993. Three-year prevalence of enteropathogenic bacteria in an urban patient population in Germany. Infection 21(2): 101-105.

MacDonald IAR, Gould IM, Curnow J. 1996. Epidemiology of infection due to Escherichia coli O157: a 3-year prospective study. Epidemiol Infect 116(3): 279-284.

Majowicz SE, Michel P, Aramini JJ, McEwen SA, Wilson JB. 2001. Descriptive analysis of endemic cryptosporidiosis cases reported in Ontario, 1996-1997. Canadian Journal of Public Health 92 (1): 62-66.

Mann ED, Sekla LH, Nayar GP, Koschik C. 1986. Infection with Cryptosporidium spp. in humans and cattle in Manitoba. Canadian journal of veterinary research 50 (2): 174-178.

Michaud S, Menard S, Arbeit RD. 2004. Campylobacteriosis, Eastern Townships, Quebec. Emerg Infect Dis 10(10): 1844-1847.

Michel P, Wilson JB, Martin SW, Clarke RC, McEwen SA, Gyles CL. 1999. Temporal and geographical distributions of reported cases of Escherichia coli O157:H7 infection in Ontario. Epidemiol Infect 122(2): 193-200.

Money P, Kelly AF, Gould SWJ, Denholm-Price J, Threlfall EJ, Fielder MD. 2010. Cattle, weather and water: mapping Escherichia coli O157:H7 infections in humans in England and Scotland. Environmental Microbiology; doi:10.1111/j.1462-2920.2010.02293.x [Online 29 May 2010].

Nakari UM, Huovinen E, Kuusi M, Siitonen A. 2010. Population-based surveillance study of Campylobacter infections in Finland. Epidemiology and Infection 138(12): 1712-1718.

Navin TR, Juranek DD, Ford M, Minedew DJ, Lippy EC, Pollard RA. 1985. Case-control study of waterborne giardiasis in Reno, Nevada. Am J Epidemiol 122(2): 269-275.

Oloya J, Theis M, Doetkott D, Dyer N, Gibbs P, Khaitsa ML. 2007. Evaluation of Salmonella occurrence in domestic animals and humans in North Dakota (2000-2005). Foodborne Pathog Dis 4(4): 551-563.

Pai CH, Ahmed N, Lior H, Johnson WM, Sims HV, Woods DE. 1988. Epidemiology of sporadic diarrhea due to verocytotoxin-producing Escherichia coli: a two-year prospective study. The Journal of Infectious Diseases 157(5): 1054-1057.

Pintar KDM, Pollari F, Waltner-Toews D, Charron DF, McEwen SA, Fazil A, et al. 2009. A modified case-control study of cryptosporidiosis (using non-Cryptosporidium-infected enteric cases as controls) in a community setting. Epidemiology and Infection 137 (12): 1789-1799.

Roy SL, DeLong SM, Stenzel SA, Shiferaw B, Roberts JM, Khalakdina A, et al. 2004. Risk factors for sporadic cryptosporidiosis among immunocompetent persons in the United States from 1999 to 2001. J Clin Microbiol 42(7): 2944-2951.

Samonis G, Maraki S, Christidou A, Georgiladakis A, Tselentis Y. 1997. Bacterial pathogens associated with diarrhoea on the island of Crete. Eur J Epidemiol 13(7): 831-836.

Sharp JC, Heymann CS. 1976. Enteric fever in Scotland, 1967-1974. J Hyg (Lond) 76(1): 83-89.

Sibbald CJ, Sharp JC. 1985. Campylobacter infection in urban and rural populations in Scotland. J Hyg (Lond) 95(1): 87-93.

Skirrow MB. 1987. A demographic survey of campylobacter, salmonella and shigella infections in England. A Public Health Laboratory Service Survey. Epidemiol Infect 99(3): 647-657.

Snel SJ, Baker MG, Kamalesh V, French N, Learmonth J. 2009. A tale of two parasites: the comparative epidemiology of cryptosporidiosis and giardiasis. Epidemiol Infect 137(11): 1641-1650.

Snel SJ, Baker MG, Venugopal K. 2009. The epidemiology of cryptosporidiosis in New Zealand, 1997-2006. New Zealand Medical Journal 122 (1290): 47-61.

Sopwith W, Ashton M, Frost JA, Tocque K, O'Brien S, Regan M, et al. 2003. Enhanced surveillance of campylobacter infection in the North West of England 1997-1999. J Infect 46(1): 35-45.

Studahl A, Andersson Y. 2000. Risk Factors for Indigenous Campylobacter Infection: A Swedish Case-Control Study. Epidemiology and Infection 125(2): 269-275.

Thomas A, Cheasty T, Frost JA, Chart H, Smith HR, Rowe B. 1996. Vero cytotoxin-producing Escherichia coli, particularly serogroup O157, associated with human infections in England and Wales: 1992-1994. Epidemiology and Infection 117(01): 1-10.

Thompson JS, Cahoon FE, Hodge DS. 1986. Rate of Campylobacter spp. isolation in three regions of Ontario, Canada, from 1978 to 1985. J Clin Microbiol 24(5): 876-878.

Toyofuku H. 2008. Epidemiological data on food poisonings in Japan focused on Salmonella, 1998-2004. Food Addit Contam Part A Chem Anal Control Expo Risk Assess 25(9): 1058-1066.

Waters JR, Sharp JCM, Dev VJ. 1994. Infection Caused by Escherichia coli O157:H7 in Alberta, Canada, and in Scotland: A Five-Year Review, 1987–1991. Clinical Infectious Diseases 19(5): 834-843.

Wielinga PR, de Vries A, van der Goot TH, Mank T, Mars MH, Kortbeek LM, et al. 2008. Molecular epidemiology of Cryptosporidium in humans and cattle in The Netherlands. International Journal for Parasitology 38 (7): 809-817.

Willshaw GA, Cheasty T, Smith HR, O'Brien SJ, Adak GK. 2001. Verocytotoxin-producing Escherichia coli (VTEC) O157 and other VTEC from human infections in England and Wales: 1995-1998. J Med Microbiol 50(2): 135-142.

Wright RA, Spencer HC, Brodsky RE, Vernon TM. 1977. Giardiasis in Colorado: an epidemiologic study. Am J Epidemiol 105(4): 330-336.

Younus M, Wilkins MJ, Arshad MM, Rahbar MH, Saeed AM. 2006. Demographic risk factors and incidence of Salmonella enteritidis infection in Michigan. Foodborne Pathog Dis 3(3): 266-273.

Zintl A, Proctor AF, Read C, Dewaal T, Shanaghy N, Fanning S, et al. 2009. The prevalence of Cryptosporidium species and subtypes in human faecal samples in Ireland. Epidemiol Infect 137 (2): 270-277.
